
JOURNAL INFORMATION
==============================
NLM Title Abbreviation: Buffalo Med J
NLM Journal ID: 0027534

Buffalo Medical Journal

ISSN: 1040-3817
Publisher: Biomedical Journal Digitization Project

ARTICLE INFORMATION
==============================
PMCID: PMC8730904
Article version: 1
Subjects: Books and Authors

A Manual of Physical Diagnosis

Print publication date: 1911 May
Volume: 66
Issue: 10
First page: 580
Last page: 580
License: This work is free of known copyright restrictions. For more information, please see PMC Back Issue Digitization.
License URL: https://creativecommons.org/publicdomain/mark/1.0/

==============================

**A Manual of Physical Diagnosis.** By **Brefney Rolph O'Reilly**, M.D., C.M. (F.T.M.C. Toronto, M.R.C.S. England, L.R.C.P. London), Demonstrator in Clinical Medicine and in Pathology, University of Toronto; Assistant Physician to St. Michael's Hospital, Toronto; Physician to Toronto Hospital for Incurables. 12mo, pp. 390. With six plates and forty-nine other illustrations. Philadelphia: P. Blakiston's Son & Company, 1012 Walnut Street. 1911.

A great deal of information is compressed in this attractive little volume on the handling and physical examination of patients. Following a short introduction, which contains some excellent hints as to management, the book covers in orderly sequence very fully the field of physical diagnosis. An appendix is added which deals with clinical pathology. The chapters on examination of lungs and heart are particularly good and up-to-date.

The book is one which can be heartily recommended to both students and practitioners. A. E. W.

---

### BOOKS RECEIVED

The Practical Medicine Series. Ten Volumes. Under the general editorial charge of Gustavus P. Head, M.D. Vol. I. General Medicine. Edited by Frank Billings, M.D. and J. H. Salisbury, M.D. Series 1911. Chicago: The Year Book Publishers. (Price, $1.50; entire series, $10.00.)

Annual Report of the Surgeon General of the Public Health and Marine-Hospital Service of the United States for the fiscal year 1910. Walter Wyman, M.D., Surgeon General.

Modern Diagnosis and Treatment of Diseases of Children. By Herman B. Sheffield, M.D., Instructor in Diseases of Children at the New York Post-Graduate Medical School and Hospital. Octavo, pp. 619. Illustrated. Philadelphia: F. A. Davis Co. 1911. (Cloth, $4.50.)

Diseases of the Stomach and Intestines. By Boardman Reed, M.D., Consulting Gastroenterologist to the Pottenger Sanatorium, Monrovia, Cal. Third edition. Octavo, pp. 1037. Illustrated. New York: E. B. Treat & Co. 1911. (Cloth, $5.00.)

Studies in the Psychology of Sex. Sex in relation to Society. Vol. VI. By Havelock Ellis. Octavo, pp. 656. Philadelphia: F. A. Davis Co. 1911. (Cloth, $3.00.)

International Clinics. A Quarterly of Illustrated Clinical Lectures and especially prepared articles on Treatment, Medicine, Surgery, Neurology, Pediatrics, Obstetrics, Gynecology, Orthopedics, Pathology, Dermatology, Ophthalmology, Otology, Rhinology, Laryngology, Hygiene, etc. Edited by Henry W. Cattell, M.D. Vol. I, twenty-first series. Philadelphia and London: J. B. Lippincott Co. 1910. (Cloth, $2.00.)

What Shall I Eat? A Manual of Rational Feeding. By Dr. F. X. Gouraud, Formerly Chief of the Laboratory of the Medical Faculty of Paris. With a Preface by Prof. Armand Gautier, of Paris. Only authorized translation by Francis J. Rebman. Octavo, pp. 379. New York: Rebman Co. 1911. (Cloth, $1.50.)

New and Nonofficial Remedies, 1911: Containing descriptions of articles which have been accepted by the Council on Pharmacy and Chemistry of the American Medical Association, prior to January 1, 1911. Pp. 282. (Paper, 25 cents; cloth, 50 cents.)

---

FOR SALE—Books, Book Cases and Furniture of the late Dr. William Warren Potter. Apply at 238 Delaware Avenue.
